# HiTaxon: a hierarchical ensemble framework for taxonomic classification of short reads

**DOI:** 10.1093/bioadv/vbae016

**Published:** 2024-02-01

**Authors:** Bhavish Verma, John Parkinson

**Affiliations:** Program in Molecular Medicine, Hospital for Sick Children, Toronto, ON M5G 0A4, Canada; Department of Molecular Genetics, University of Toronto, Toronto, ON M5S 1A8, Canada; Program in Molecular Medicine, Hospital for Sick Children, Toronto, ON M5G 0A4, Canada; Department of Molecular Genetics, University of Toronto, Toronto, ON M5S 1A8, Canada; Department of Biochemistry, University of Toronto, Toronto, ON M5S 1A8, Canada

## Abstract

**Motivation:**

Whole microbiome DNA and RNA sequencing (metagenomics and metatranscriptomics) are pivotal to determining the functional roles of microbial communities. A key challenge in analyzing these complex datasets, typically composed of tens of millions of short reads, is accurately classifying reads to their taxa of origin. While still performing worse relative to reference-based short-read tools in species classification, ML algorithms have shown promising results in taxonomic classification at higher ranks. A recent approach exploited to enhance the performance of ML tools, which can be translated to reference-dependent classifiers, has been to integrate the hierarchical structure of taxonomy within the tool’s predictive algorithm.

**Results:**

Here, we introduce HiTaxon, an end-to-end hierarchical ensemble framework for taxonomic classification. HiTaxon facilitates data collection and processing, reference database construction and optional training of ML models to streamline ensemble creation. We show that databases created by HiTaxon improve the species-level performance of reference-dependent classifiers, while reducing their computational overhead. In addition, through exploring hierarchical methods for HiTaxon, we highlight that our custom approach to hierarchical ensembling improves species-level classification relative to traditional strategies. Finally, we demonstrate the improved performance of our hierarchical ensembles over current state-of-the-art classifiers in species classification using datasets comprised of either simulated or experimentally derived reads.

**Availability and implementation:**

HiTaxon is available at: https://github.com/ParkinsonLab/HiTaxon.

## 1 Introduction

Microbiota in the human gut are increasingly being shown to play a key role in health and disease (Hou *et al.* 2022). To understand how they contribute to disease, attention has turned to interrogating their functional capacity through whole microbiome DNA (“metagenomics”) and RNA (“metatranscriptomics”) sequencing (Chung *et al.* 2020), primarily using short-read next-generation sequencing technology (Shakya *et al.* 2019). A critical component of these analyses is assigning sequenced reads to their taxa of origin (Beghini *et al.* 2021, Taj *et al.* 2023). Traditional reference-dependent tools used for taxonomic analysis can be categorized into marker-based, DNA-based, or protein-based approaches (Ye *et al.* 2019). In the former, taxon-specific marker genes are used to generate a taxonomic profile of the target dataset (Ye *et al.* 2019). In the latter two methods, reads are mapped to reference databases of DNA or protein sequences for taxonomic classification (Ye *et al.* 2019), thereby informing on the contributions of individual taxa to microbiome function.

One of the best performing DNA-based tools is Kraken2 (Wood *et al.* 2019), a k-mer based approach where each k-mer from a query read is assigned to the lowest common ancestor (LCA) associated with the set of genomes within its reference database that retain that k-mer. A major challenge for Kraken2 and other reference-based approaches is the choice of database used for sequence matching. For example, the LCA scheme used by Kraken2, which improves the precision of predictions, was found to hinder species classification when Kraken2’s prior variant was paired with large reference databases (Nasko *et al.* 2018). This issue arises due to the presence of a large number of identical sequences in related taxa (Nasko *et al.* 2018), which will worsen as reference databases continue to grow annually (Sayers *et al.* 2022). These problems are further confounded by the overrepresentation of a limited number of taxa. For instance, 90% of the 639 981 high-quality bacterial assemblies in the European Nucleotide Archive (as of November 2018) were associated with only 20 unique species (Blackwell *et al.* 2021). This biased distribution, in turn, can be a frequent source of false positives (McIntyre *et al.* 2017). To mitigate the effects of this compositional bias on taxonomic classification, recent work has shown that creating custom databases that better align with the taxonomic identity of the environment of interest improves the performance of Kraken2 (Smith *et al.* 2022). Together these findings establish the need for a framework capable of systematically curating custom nonredundant databases for reference-dependent tools appropriate for the dataset being analyzed.

As an alternative to reference-dependent classifiers, researchers have explored the application of classical machine learning algorithms such as Naïve Bayes and Support Vector Machines (Rosen *et al.* 2008, Vervier *et al.* 2016) alongside modern deep learning architectures previously applied to computer vision (Cres *et al.* 2023) and natural language processing (Liang *et al.* 2020, Mock *et al.* 2022) for taxonomic classification. While these ML approaches perform worse in species-level assignments when compared to reference-dependent classifiers (Vervier *et al.* 2016, Liang *et al.* 2020, Cres *et al.* 2023), they have demonstrated comparable or even superior performance at higher ranks of taxonomy (Liang *et al.* 2020, Shang and Sun 2021, Mock *et al.* 2022, Cres *et al.* 2023). An important innovation driving the improvement of a subset of these classifiers is the integration of taxonomic structural hierarchy through ensembling multiple classifiers trained for different parent-to-child relationships (Shang and Sun 2021, Li *et al.* 2023). While ensembling (Jiang *et al.* 2017, McIntyre *et al.* 2017, Mock *et al.* 2022) has been previously exploited for metagenomic taxonomic classification, the application of a hierarchical ensemble for species-level classification of short reads has not been thoroughly explored.

**Figure 1.**
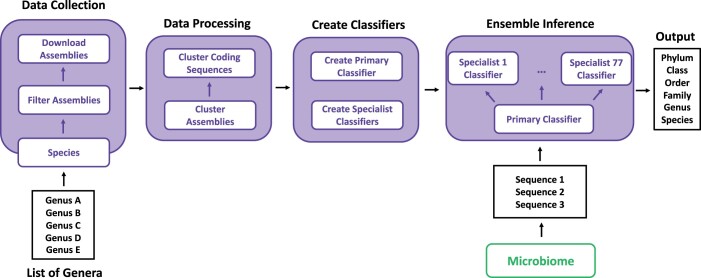
Overview of HiTaxon. Given a list of genera, HiTaxon uses pre-set filters to download high-quality assemblies for species encompassed within the set of defined genera. A two-step clustering approach is used to reduce redundant genomic information, creating species-specific nonredundant sets of coding sequences. These sets of sequences are used to create a hierarchical ensemble composed of a primary classifier and classifiers specialized for discriminating between species within a particular genus. This ensemble is used to facilitate taxonomic classification of metagenomic and metatranscriptomic reads.

Here, we present HiTaxon, an end-to-end hierarchical ensemble framework designed to accurately perform species-level classification. By streamlining data collection and processing, database construction, and optional ML model training, HiTaxon generates custom ensembles appropriate for the dataset of interest (Fig. 1). We show that databases constructed by HiTaxon improved species-level assignments while also dramatically reducing computational overhead. Adopting a custom hierarchical ensemble architecture, we further demonstrated improved species classification over pre-existing hierarchical ML strategies. Finally, we show that hierarchical ensembles improved species prediction relative to other classifiers on both simulated and experimental reads.

## 2 Methods

### 2.1 Collection and processing of reference sequences

To create sets of nonredundant sequences for reference database construction or model training, HiTaxon filters RefSeq (accessed 24 August 2022) for all assemblies encompassed within a query set of genera. On a per-species basis, HiTaxon compiles a list of assemblies, prioritizing by the following categories of assembly completeness: complete genome, chromosome, scaffold and contig. For each category, HiTaxon uses the following RefSeq labels as a secondary attribute for prioritizing assemblies: reference genome, representative genome, and NA. Using this priority-based ordering, HiTaxon aims to generate species-specific lists composed of 10 high quality assemblies. In instances where a species is represented by fewer than 10 assemblies in RefSeq, all assemblies are added to the list. However, when a species is represented by >10 assemblies, the lowest quality assemblies (i.e. those with the worst combination of assembly level and RefSeq category) that represent strains for which better quality assemblies have been obtained are excluded. Coding sequences associated with this list of high-quality assemblies are downloaded using the NCBI Datasets command-line tool (Sayers *et al.* 2022) (v. 15.3.1). Post-download, HiTaxon uses fastANI (Jain *et al.* 2018) (v. 1.33) to compute the average nucleotide identities (ANI) of an assembly against all other assemblies pertaining to the same species. Assemblies are then grouped based on ANI scores using the OPTICS clustering algorithm (Ankerst *et al.* 1999, Pedregosa *et al.* 2011) (scikit-learn v. 1.1.3). To determine the optimal set of parameters for the OPTICS algorithm, the average silhouette coefficient (Rousseeuw 1987) was computed from individual silhouette coefficients calculated for species-specific clusters generated for the human gut HiTaxon database. This involved testing 10 different combinations of the parameters: min_samples, max_eps, and metric (Supplementary Data S1). We found that setting min_samples to two and leaving the remaining parameters at their default values was one of two combinations that yielded the highest average silhouette coefficient. Thus, for all HiTaxon databases referenced throughout the text, this parameter combination was used. Adopting this ANI mediated unsupervised clustering approach avoids inconsistencies that can otherwise arise when relying only on a nomenclature based approach (Parks *et al.* 2018). For each group of assemblies generated by OPTICS, the assembly with the highest N50 score, as provided by RefSeq, is selected as the representative assembly. Subsequently, HiTaxon aggregates individual coding sequences into a single species-specific FASTA file. CD-HIT-EST (Li and Godzik 2006) (v. 4.8.1) is then applied to the aggregated FASTA file to cluster individual coding sequences sharing 99% sequence identity, creating a species-specific dataset of nonredundant coding sequences.

### 2.2 Hierarchical frameworks

**Figure 2.**
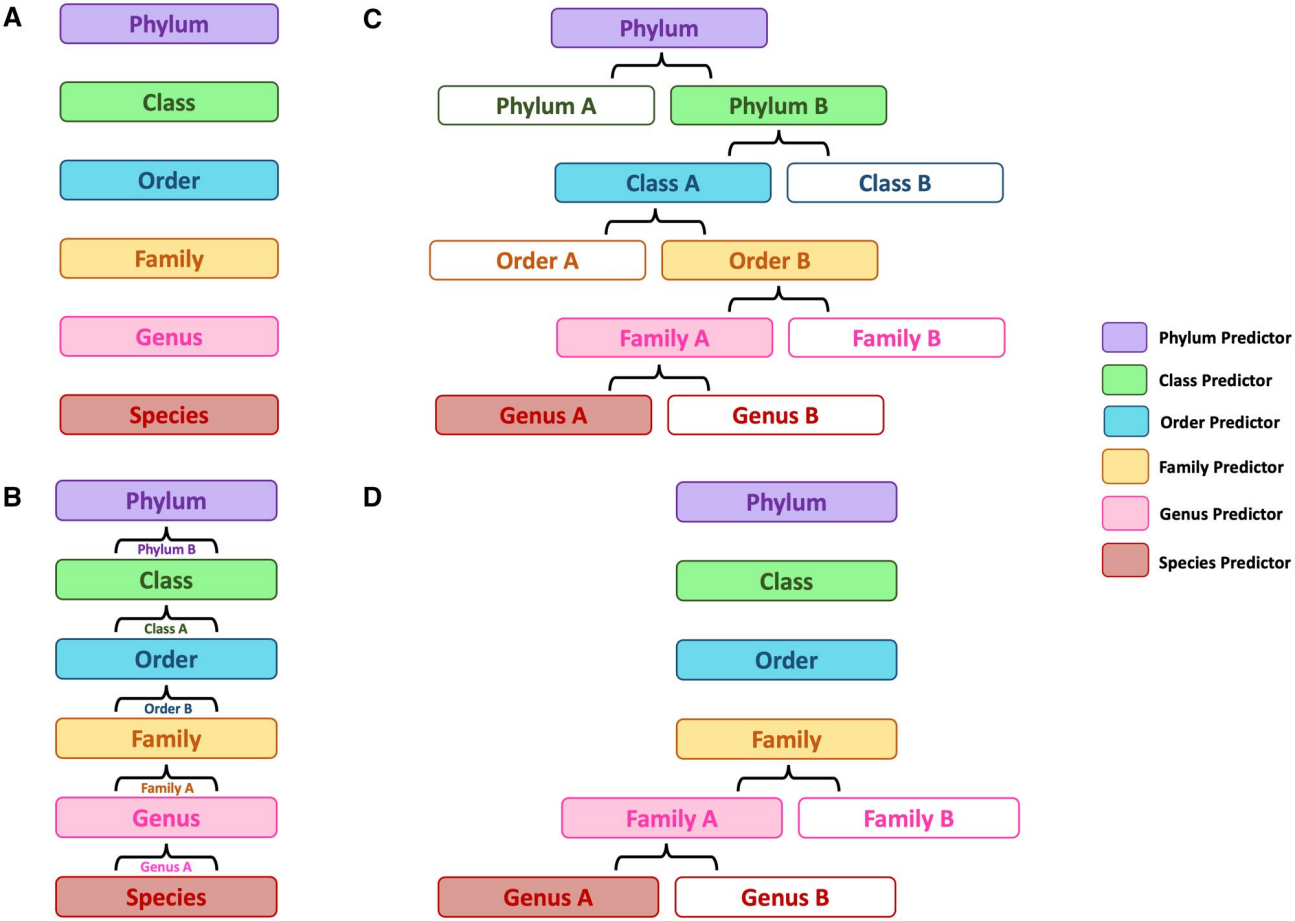
Hierarchical machine learning methods for species classification. (A) The local classifier per level (LCL) approach uses classifiers trained for a specific taxonomic rank to generate predictions at that level. (B) The hierarchy-informed LCL approach uses the same classifiers used in the LCL strategy, but the outputs derived from classifiers at a higher taxonomic rank are used to minimize the subset of predictions considered from the classifier corresponding to the subsequent lower rank. For instance, if the *Phylum* classifier were to predict the read as being from *Phylum B*, then for the subsequent prediction generated by the *Class* classifier, only *Classes* encompassed within *Phylum B* would be considered. This is repeated until a *Species* prediction is generated. (C) The local classifier per parent node (LCPN) method uses predictions generated by classifiers at higher taxonomic ranks to determine which child classifier to use at lower taxonomic levels. For instance, if the *Phylum* classifier were to predict the read as being from *Phylum B*, then the *Phylum B* specific classifier would be used over the *Phylum A* classifier to predict the *Class* in which the read is derived from. This is repeated until a *Species* prediction is generated. (D) The LCL-LCPN method is a custom hybrid approach which uses the LCL strategy from *Phylum-to-Family* and the LCPN protocol from *Genus-to-Species*.

Given that hierarchical ML ensembles have shown promise for improving classification performance (Shang and Sun 2021, Li *et al.* 2023), we investigated four hierarchical frameworks (Fig. 2). First, a local classifier per level (LCL) approach (Silla and Freitas 2011), which uses a single classifier for each taxonomic level. Second, a hierarchy-informed LCL approach (Silla and Freitas 2011), which uses the predictions of a higher taxonomic rank classifier to determine which subset of outputs generated by the subsequent lower-rank classifier are considered. Note that this approach uses the same classifiers used in the LCL approach. Third, a local classifier per parent node (LCPN) approach (Silla and Freitas 2011), which integrates multi-class classifiers for each taxon across all taxonomic ranks and uses the predictions of a higher taxonomic rank model to determine which child classifier is used for predictions at that subsequent lower rank. Fourth, a hybrid of the LCL and LCPN approach (LCL-LCPN), where the LCL method is used for phylum-to-family level classification, while the LCPN method is used for genus and species level predictions. We note that the LCL approach has previously been used for species-level classification (Vervier *et al.* 2016). However, prior applications of the LCPN approach to taxonomic classification were either restricted to higher taxonomic ranks (Shang and Sun 2021) or relied on larger genomic fragments as input (Li *et al.* 2023). To our knowledge, neither the hierarchy-informed LCL approach nor the hybrid LCL-LCPN approach has been used for the purposes of taxonomic classification.

Since previous applications of hierarchical frameworks were ML-based, we decided to use a supervised ML algorithm to compare the four hierarchical approaches. In particular, a CPU-efficient NLP word embedding algorithm called FastText (Joulin *et al.* 2017) (v. 0.9.2) was used. In addition to its competitive performance with convolutional and recurrent neural networks, FastText has a shorter training time, rendering it better suited to the multi-classifier nature of local hierarchical architectures. Furthermore, a prior application of FastText to taxonomic classification reported competitive performance compared to an older alignment algorithm (Menegaux and Vert 2019).

### 2.3 Ensembles

In addition to hierarchical ensembles composed solely of ML classifiers, we also investigated the performance of hierarchical frameworks composed either entirely of reference-dependent tools or a combination with ML classifiers. Note that for all classifiers integrated into an ensemble, their reference sequences or training data were derived from HiTaxon curated sequences. Ensembles with both ML and reference-dependent classifiers used either one of two state-of-the-art reference-dependent tools, Kraken2 (Wood *et al.* 2019) (v. 2.1.2) or Kaiju (Menzel *et al.* 2016) (v. 1.9.2), and are referred to as Kraken2-HiTaxon-ML and Kaiju-HiTaxon-ML respectively. Within these ensembles, Kraken2 or Kaiju functioned as the primary classifier in which their phylum to genus-level predictions were used. The genus-level outputs of the primary classifier guided the selection of specialized ML classifiers. These specialized classifiers were focused on differentiating between species present within the particular genus identified by the primary classifier, thereby generating classifications at the species-level. If an ML prediction had a softmax score <0.5, the lowest taxonomic rank output of the reference-dependent tool was used instead. If no prediction was provided by the reference-dependent tool for a particular read, it was left unclassified. We also tested a similar hierarchical ensemble using Kraken2 and BWA (Li and Durbin 2009), referred to as Kraken2-HiTaxon-Align, in which specialized BWA indices for species classification were built for each genus. As for the previous ensemble, Kraken2 functioned as the primary classifier, where its genus-level outputs determined what genus-specific BWA indices were used for mapping. If BWA mapped a single read to multiple references, only the reference with the highest alignment score, and the species corresponding to that reference, was retained. In instances where a read was left unmapped or had the same maximum alignment score for multiple references pertaining to different species, the lowest taxonomic rank prediction of Kraken2 was used instead. Furthermore, to compare hierarchical ensembles relative to an alternative approach for ensembling, we combined the outputs of Kaiju and Kraken2 using a more standard strategy, which we refer to as Kraken2-Kaiju-Ens. Within this approach, if Kraken2 could not generate a species-level output, the species-level prediction of Kaiju was used instead. If neither approach generated a species-level output, Kraken2’s lowest rank prediction was used. Note that when used for short-read classification, besides number of threads used, Kraken2’s, Kaiju’s, and BWA’s parameters were set to their default values for all evaluations.

### 2.4 Classifier evaluations

To evaluate the performance of individual classifiers and ensembles we simulated multiple datasets that correspond to the genus-level identity of different microbiome environments (human gut, human conjunctival, marine surface, rumen). To create these datasets, we used sequences exclusive to GenBank, including those suppressed from RefSeq. As a result, at no point were these assemblies seen in the HiTaxon pipeline, ensuring no data leakage. Note: For the rumen dataset, cultivated genomes (Seshadri *et al.* 2018) and metagenome assembled genomes (Stewart *et al.* 2019) were used to simulate reads. In addition, we also tested the performance of each classifier on gold-standard experimental datasets of DNA and RNA reads, where the taxa responsible for the reads were known (refer to Table 1 and Supplementary Text S1 for detailed information on creating simulated and experimental datasets).

**Table 1.** Characteristics of the training and test data used to build and evaluate taxonomic classifiers.

Dataset	No. of training genera	No. of training species	No. of HiTaxon-DB coding sequences	No. of RefSeq-DB coding sequences	No. of test species	No. of test reads	Simulated
Human gut	58	1718	12 027 988	269 431 618	522	3 415 471–3 605 377	Yes
Human conjunctival	10	780	5 487 293	NA	256	1 996 771–2 110 540	Yes
Marine surface	43	1651	11 358 799	182 996 521	337	2 867 107–3 083 814	Yes
Rumen	NA	NA	NA	NA	NA	1 159 325	Yes
Mock community 1	8	646	6 018 839	205 425 935	8	4 556 549	No
Mock community 2	23	1275	10 863 883	389 602 189	28	2 599 072	No

To create custom Kraken2 and Kaiju HiTaxon databases (Kraken2-HiTaxon-DB and Kaiju2-HiTaxon-DB) suitable for a specific dataset, we used species-specific HiTaxon curated coding-sequences encompassed in the genera relevant to that specific dataset (Table 1). In addition, for most datasets, we also created databases for both Kraken2 and Kaiju (Kraken2-RefSeq-DB and Kaiju2-RefSeq-DB), using all assemblies from RefSeq downloaded by the NCBI Datasets tool for a set of dataset-specific species. This approach mimics an ad-hoc method to database construction (Table 1). Note: For 8 of the 3331 species used in our evaluations (combining all simulated and experimental datasets), we limited their contribution to 5000 assemblies in the RefSeq-DB databases to ensure that database construction runtimes did not exceed 24 h (see Supplementary Text S1 for specific species). For both HiTaxon-DB and RefSeq-DB, only sequences pertaining to NCBI formal named species adhering to the international code of nomenclature of prokaryotes (ICNP) were used. Since Kaiju is a peptide-based classifier, we used Prodigal (Hyatt *et al.* 2010) to perform 6-frame translations of the reference nucleotide sequences.

To train the ML classifiers for the hierarchical frameworks, we derived 500 000 150 bp paired-end ART (Huang *et al.* 2012) (v. 2.5.8) simulated reads, for each species, from the same set of HiTaxon species-specific coding-sequences used to build the HiTaxon-DB databases. Prior to training, we aggregated, merged, shuffled, and tokenized reads into 13-mers. During the generation of ML classifiers for the human conjunctival dataset, we used 464 RefSeq assemblies, not selected for training, to create a validation set composed of 1 221 740 reads. This validation set was then used to select optimal hyperparameters for the LCL species-level FastText classifier, through a random search approach (Bergstra and Bengio 2012). Based on this search, we found that a FastText model with an embedding dimension of 200, a context window of 5, wordNgrams of 1, and a learning rate of 0.5, trained for 15 epochs and evaluated using a confidence threshold of 0.5, provided the best species-level performance. The same set of hyperparameters (excluding learning rate and number of epochs to keep training under 24 h) were used for the remaining classifiers and subsequent datasets to best simulate the application of HiTaxon using default settings.

To assess classifier performance, the primary metric we used was the Multi-Class Matthews Correlation Coefficient (Gorodkin 2004) (MCC):

$$\mathrm{MCC}=\;(\frac{c\;\times \;s-\;\sum_{k}^{K}p_{k}\times \;t_{k}\;}{\sqrt{\left(s^{2}-\sum_{k}^{K}p_{k}^{2}\right)\times (s^{2}-\sum_{k}^{K}t_{k}^{2})}})$$

MCC can be defined in terms of a confusion matrix *C* for *K* classes (Pedregosa *et al.* 2011):

t_*k*_ = $\sum_{i}^{K}C_{ik}\;$the number of times class *k* truly occurred.

p_*k*_ = $\sum_{i}^{K}C_{ki}\;$ the number of times class *k* was predicted.

c *=* $\sum_{k}^{K}C_{kk}\;$the total number of samples correctly predicted.

s = $\sum_{i}^{K}\sum_{j}^{K}C_{ij}\;$the total number of samples.

### 2.5 Computing environment

Classifiers were created and benchmarked on the SciNet operated Niagara compute cluster, using compute nodes with 40 Intel Skylake cores @ 2.40 GHz with 202 GB of RAM running CentOS 7. We also measured the runtimes of pre-built HiTaxon-based classifiers on a 2.6 GHz 6-core Intel Core i7 MacBook Pro with 16 GB of RAM when evaluating reads from Mock Community 1 and 2.

## 3 Results

### 3.1 Overview of HiTaxon

Here, we were interested in addressing two major concepts associated with taxonomic classification accuracy. First, the reference database used for taxonomic assignments, given its known capacity to significantly impact classifier performance. Second, the use of a hierarchical ensemble to improve species classification. This approach also allows for the assignment of labels to higher ranks for lower confidence predictions, thereby helping to reduce false positives. To integrate these concepts, we developed the HiTaxon framework (Fig. 1). For classifier construction within HiTaxon, researchers need to provide a list of genera relevant to their specific environment of interest. In environments with extensive experimental studies, researchers could utilize a union of genera-level taxonomic identities described in prior metagenomic studies. However, even within well-studied microbiomes, this approach could miss important genera specific to the researcher’s dataset. Therefore, in instances where complementary 16S rDNA sequence survey data is available, integrating the genera identified from the 16S dataset with information derived from the work of prior researchers is potentially a more comprehensive approach. In cases where complementary 16S data is unavailable, marker-based taxonomic approaches could instead be used to generate a pre-defined list of genera for HiTaxon. For each species encompassed within these genera, the HiTaxon framework automatically collates a set of nonredundant coding sequences. These sequences are then used to construct an appropriate custom reference database for a primary classifier to facilitate phylum-to-genus predictions. Following this, HiTaxon creates multiple specialized species-level classifiers for each input genus. Finally, HiTaxon uses a hierarchical classification algorithm to integrate the predictions of the primary classifier together with the specialized classifiers. In the following, we assess the performance of the HiTaxon framework, with respect to both its approach to generating custom databases and its use of hierarchical ensembles.

### 3.2 Databases with nonredundant sequences elevate reference-dependent species classification

Key to the performance of any reference-based classifier is the generation of an appropriate reference database. Given a list of genera, HiTaxon generates sets of nonredundant coding sequences for all encompassed NCBI formal named species.

We benchmarked the performance of HiTaxon’s systematic database curation approach by pairing Kraken2 (Wood *et al.* 2019) and Kaiju (Menzel *et al.* 2016), with either HiTaxon curated sequences (HiTaxon-DB) or a custom database composed of unprocessed RefSeq sequences pertaining to the same subset of species in HiTaxon-DB (RefSeq-DB). We applied these classifiers to taxonomically assign simulated sequence reads. Classifiers using the RefSeq-DB and HiTaxon-DB databases were built with, respectively, 67 490 and 8407 assemblies for 1718 species encompassed within 58 genera associated with the human gut microbiome (King *et al.* 2019) (Table 1). Using 1641 GenBank assemblies that comprise of 522 species encompassed in the same 58 genera, we simulated 10 unique test sets that ranged between 3 415 471 and 3 605 377 paired-end reads.

**Figure 3.**
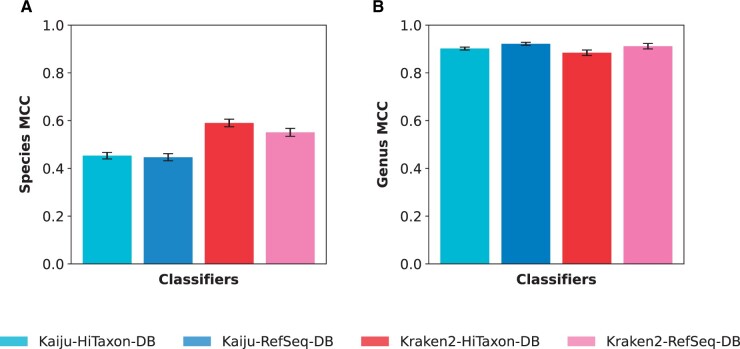
Performance of Kaiju and Kraken2 with HiTaxon databases. Using simulated test sets consistent with the genera-level identity of the human gut microbiome, the (A) species- and (B) genus-level MCC was computed for classifiers with the RefSeq-DB database and the HiTaxon-DB database.

Focusing on species-level performance (Fig. 3A), we found that when using the HiTaxon database, Kaiju and especially Kraken2 outperformed their counterparts using the RefSeq-DB database in species classification by an average of 1.45% ± 0.69 and 7.13% ± 1.26 in MCC respectively (Wilcoxon signed-rank test with Benjamini-Hochberg correction: *P* < 0.05). Performance gains were largely driven through fewer unclassified reads; Kraken2 and Kaiju assigned 9.35% ± 0.56 and 10.23% ± 0.43 more reads, respectively, using the HiTaxon-DB database. These results highlight previous findings in which the additional noise introduced with redundant sequences compromises the ability of tools that utilize LCA algorithms to classify reads at the species-level (Nasko *et al.* 2018).

With respect to genus classification (Fig. 3B), however, both Kraken2-HiTaxon-DB and Kaiju-HiTaxon-DB exhibited slightly poorer performance relative to when using the RefSeq-DB database, decreasing MCC by an average of 2.04% ± 0.08 and 1.23% ± 0.32, respectively. Unlike species-level classification, we found that Kraken2-RefSeq-DB and Kaiju-RefSeq-DB left fewer reads unclassified at the genus-level when compared to the HiTaxon-DB databases (1.68% ± 0.081 and 0.59% ± 0.41 additional reads identified using Kraken2-RefSeq-DB and Kaiju-RefSeq-DB, respectively). These observations recapitulate prior results which suggest that as databases grow, species-level classification performance decreases, while genus-level classification accuracy increases (Nasko *et al.* 2018). This trend likely occurs as a consequence of larger databases containing a greater number of redundant sequences. Such additional sequences would provide Kraken2 and Kaiju more opportunities for read matching, albeit to multiple taxa. Consequently, the LCA algorithms used by these two tools would encourage the classifiers to generate higher-taxonomic predictions, again highlighting the sensitivity of LCA schemes to reference databases. This marginal improvement extended to other higher taxonomic ranks (Supplementary Data S1). Interestingly, the narrowing of performance between Kaiju and Kraken2 from species to genus classification likely reflects the ability of synonymous SNPs in DNA at better discriminating species.

Beyond improved species-classification, HiTaxon provides substantial computational benefits when building custom databases (Table 2). RefSeq-DB databases required approximately 5× and 19× more memory for Kraken2 and Kaiju, respectively, compared to HiTaxon-DB databases. In addition, it was approximately 21× and 28× faster to build HiTaxon-DB databases for Kaiju and Kraken2, respectively, relative to RefSeq-DB databases.

**Table 2.** Computing resources used by Kaiju and Kraken2 with RefSeq and HiTaxon databases.

Classifier	Database	Build Time	Memory
Kaiju	RefSeq-DB	6 h and 31 min	210 GB
Kaiju	HiTaxon-DB	18 min	11 GB
Kraken2	RefSeq-DB	13 h and 27 min	333 GB
Kraken2	HiTaxon-DB	29 min	63 GB

### 3.3 A hybrid approach to hierarchical ensembling improves species-level performance

Having demonstrated the improved performance of using HiTaxon’s custom database for species-level classification with existing algorithms, we next focused on how hierarchical ensembling could benefit predictive performance. We investigated three traditional hierarchical ML ensembling strategies: LCL, Hierarchy-informed LCL, and LCPN (Fig. 2). In addition, we also tested a custom approach: LCL-LCPN. To compare hierarchical strategies, we trained each approach to classify 780 species belonging to 10 genera associated with the human conjunctival microbiome (Huang *et al.* 2016). Species-specific HiTaxon curated coding sequences used to train these architectures were obtained from 3648 RefSeq assemblies (Table 1). The benchmarking of each approach was evaluated using 10 unique test sets, ranging between 1 996 771 and 2 110 540 paired-end reads, derived from 767 GenBank assemblies associated with 256 species encompassed in the human conjunctival genera. In addition, we also built a HiTaxon-DB database for Kraken2, the best performing species classifier in the previous test, using the same set of HiTaxon curated coding sequences utilized for training the ML models.

Comparing the four hierarchical strategies (Table 3), the hybrid LCL-LCPN approach performed the best in terms of species-level assignments (Wilcoxon signed-rank test with Benjamini-Hochberg correction: *P* < 0.05, with respect to the next best performing classifier). When comparing the performance of the LCPN and LCL-LCPN methods at the family-level, recalling that the hybrid LCL-LCPN approach uses the LCL classifier for family predictions, we found that the LCL-LCPN’s accuracy was 2.23% ± 0.07 greater on average (Supplementary Data S2). This reduced performance at the family level by the LCPN method suggests that compounding errors from incorrect predictions at higher ranks outweigh the benefit of using hierarchy-informed predictions at all taxonomic levels. This suggestion is further strengthened when comparing the species-level performance of the two LCL approaches. Despite using the same trained classifiers, the hierarchy-informed LCL method had an average 1.64% ± 0.08 lower MCC at the species-level relative to the conventional LCL strategy (Table 3). An additional benefit to using the LCL-LCPN approach over the LCPN strategy is that it required training 14 fewer models (Table 3). We found that the two closest classifiers in species-level performance, with an average difference of 0.0038 ± 0.003 in their MCC, was the LCL-LCPN approach and Kraken2 (Table 3). These results highlight that the trained ML classifiers, when ensembled using an appropriate hierarchical strategy, can be competitive with current state-of-the-art reference-dependent tools.

**Table 3.** Performance of the four hierarchical strategies and Kraken2 on simulated human conjunctival datasets.

Classifier	Species MCC	No. of models needed
LCL	0.547 ± 0.018	6
Hierarchy-Informed LCL	0.538 ± 0.018	6
LCPN	0.585 ± 0.017	37
LCL-LCPN	0.592 ± 0.017	23
Kraken2-HiTaxon-DB	0.596 ± 0.015	1

### 3.4 A hierarchical ensemble with Kraken2 outperforms all other classifiers on simulated reads

Having created a novel hierarchical ML ensemble that appears competitive with Kraken2, we reasoned that since the two methods utilize different algorithms, their combination might lead to further performance gains. We therefore generated a hierarchical ensemble with a similar approach to the LCL-LCPN method. To minimize the compute time required to train phylum to genus ML models, we used Kraken2 for phylum-to-genus predictions. Kraken2’s genus outputs were used to determine which species-level ML classifiers to use for predictions. We opted to use ML classifiers as the primary species predictor within our hierarchical ensemble to mitigate classification biases associated with over-represented species in reference databases (McIntyre *et al.* 2017). Despite HiTaxon using a systematic approach to reduce redundant data, we still found that our custom databases for reference-dependent tools resulted in an uneven distribution of sequence data for individual species. For instance, we used 250 605 coding sequences for *Escherichia coli* and only 4253 sequences for *Escherichia whittamii* in our human gut HiTaxon database. HiTaxon still provides a significant removal of bias when we consider that our custom human gut RefSeq database comprises of 25 332 170 and 8509 coding sequences for *E.coli* and *E.whittamii*, respectively. However, prior to training species-level ML classifiers, we balanced our training set such that an approximately equal number of simulated reads from HiTaxon curated species-specific sequences were used for training; thus ensuring models were not biased toward certain species within particular genera. In instances, where the ML classifier reported low quality predictions (softmax score < 0.5), Kraken2’s predictions were used instead, regardless of the prediction’s taxonomic level (see Section 2 for more detail).

In addition to evaluating the performance of the ensemble (Kraken2-HiTaxon-ML) against Kraken2, Kaiju and the LCL-LCPN method, we also investigated two other ensembles involving Kaiju predictions (see Section 2 for Kaiju-HiTaxon-ML and Kraken2-Kaiju-Ens). Classifiers were constructed from sequence data pertaining to 1651 species present in 43 genera associated with the marine surface microbiome (Keller *et al.* 2021). Two custom reference databases were used, HiTaxon-DB (4770 assemblies) and RefSeq-DB (43 807 assemblies) (Table 1). Unlike, the RefSeq-DB database used for the human gut microbiome, we built the marine surface RefSeq-DB database without limiting any species to 5000 RefSeq assemblies to determine whether this threshold negatively influenced classifiers using RefSeq-DB. Despite this, the RefSeq-DB marine surface microbiome database used 35.1% fewer RefSeq assemblies when compared to the human gut microbiome database even though it only encompassed 3.9% fewer species (1651 species versus 1718); highlighting the reduced coverage of organisms used in the marine surface microbiome simulation. 10 unique test sets composed of 337 species encompassed in the 43 genera used for building reference databases, ranging between 2 867 107 and 3 083 814 paired-end reads, were derived from 974 GenBank assemblies and used to compare classifiers.

**Figure 4.**
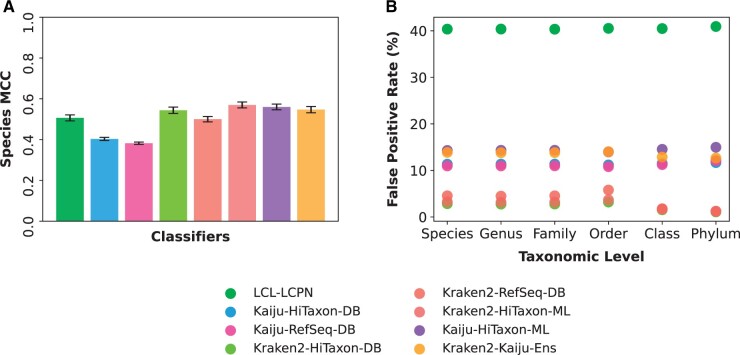
Performance of classifiers on simulated datasets. (A) Using simulated test sets consistent with the genera-level identity of the marine surface microbiome, the species-level MCC was computed for each classifier. (B) At each taxonomic rank, the proportion of simulated rumen microbiome reads pertaining to taxa absent in the marine surface simulation that were inappropriately assigned a species label was measured.

As before, both Kaiju-HiTaxon-DB and Kraken2-HiTaxon-DB improved species MCC by 5.5% ± 2.2 and 8.72% ± 2.6, respectively, relative to the RefSeq-DB database; illustrating the robustness of HiTaxon’s data reduction framework (Fig. 4A). Unlike the comparisons pertaining to the simulated human conjunctival microbiome sequences, Kraken2-HiTaxon-DB (0.544 MCC ± 0.015) comfortably outperformed the LCL-LCPN (0.506 MCC ± 0.014) approach, suggesting that the LCL-LCPN approach may be compromised by complex datasets. We also tested Kraken2-RefSeq-DB and Kraken2-HiTaxon-DB using a more stringent confidence threshold of 0.1 and found that, as expected, it improved precision but also reduced the number of true positives identified such that the MCC decreased by 3.08 ± 0.322 and 3.37 ± 0.272, respectively, relative to default parameters (Supplementary Data S3). Focusing on the ensemble classifiers, we found all three exhibited superior performance to any single classifier, with Kraken2-HiTaxon-ML (0.57 MCC ± 0.014) performing the best (Wilcoxon signed-rank test with Benjamini-Hochberg correction: *P* < 0.05, with respect to the next best performing classifier), followed by Kaiju-HiTaxon-ML. When compared to the Kraken2-RefSeq-DB baseline, Kraken2-HiTaxon-ML provides a 13.96% ± 2.3 improvement in species MCC.

### 3.5 HiTaxon-based Kraken2 classifiers minimize false positives on unseen taxa

Previous estimates suggest only 2.1% of prokaryotic taxa have been sequenced (Zhang *et al.* 2020), which implies classifiers may be confounded by species not present in their reference databases. This has the potential to increase the incidence of false positives. This motivated our investigation into the susceptibility of each classifier to false positives, using reads derived from taxa absent in the marine surface microbiome reference database.

We simulated a rumen microbiome test set from a collection of 349 rumen cultivated genomes (Seshadri *et al.* 2018) and rumen microbiome metagenome assembled genomes (Stewart *et al.* 2019). This test set consisted of 1 159 325 paired-end reads, where the majority of the reads pertained to 113 species, 57 genera, 33 families, 22 orders, 11 classes, and 6 phyla absent in the marine surface microbiome simulation. For each taxonomic rank, reads in the rumen microbiome test set assigned to taxa at that rank which were absent in the marine surface microbiome simulation were isolated for. Using these subsets of reads, we measured the amount of species-level false positives that were generated by classifiers when encountering unseen taxa across all ranks (Supplementary Data S4). Overall, we found the proportion of false positives generated by classifiers was relatively consistent across all taxonomic levels (Fig. 4B). Relative to other classifiers, the LCL-LCPN approach was most prone to generating false positives (40.5% ± 0.19 for all taxonomic ranks). Kraken2-HiTaxon-DB performed the best, with an average of 2.37% ± 0.774 for all taxonomic ranks, indicating that the poor performance of the LCL-LCPN algorithm is not a consequence of HiTaxon’s data collection and processing. We found that Kraken2-HiTaxon-ML (average of 3.715% ± 0.893 for all taxonomic ranks) was second only to Kraken2-HiTaxon-DB in avoiding false positives, performing better then Kraken2-Kaiju-Ens (average of 13.5% ± 0.532 for all taxonomic ranks).

### 3.6 A hierarchical ensemble of reference-dependent classifiers improves species classification of experimental reads

To ensure the improved performance derived from our approach to database construction and ensembling translates to experimentally derived reads, we investigated classification performance on two previously published experimentally derived datasets, termed “Mock Community 1” and “Mock Community 2” described below. In addition, given the superior performance of our hierarchical ensemble of Kraken2 and ML models in species classification over alternative approaches, we were interested in understanding whether a hierarchical ensemble composed entirely of reference-dependent classifiers would provide similar benefits. Thus, we opted to create a hierarchical ensemble of Kraken2 and BWA (see Section 2). As akin to the reasoning to combine Kraken2 and ML models, the alignment algorithm of BWA is sufficiently different from Kraken2’s k-mer matching strategy such that it could generate an effective ensemble. We refer to this hierarchical ensemble as Kraken2-HiTaxon-Align.

**Figure 5.**
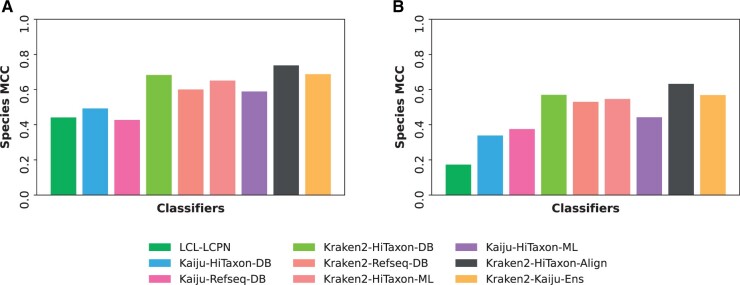
Performance of classifiers on experimental datasets. For both (A) Mock Community 1 and (B) Mock Community 2, which are composed of experimentally derived reads, species-level MCC was computed.

The first dataset, Mock Community 1, comprised 4 556 549 paired-end reads, and was generated from a sample composed of the eight bacterial taxa associated with the ZymoBIOMICS Microbial Community Standard (Nicholls *et al.* 2019). Classifiers were created using 6 018 839 HiTaxon curated coding sequences generated from 5463 assemblies (646 species) that pertain to the eight bacterial genera associated with the ZymoBIOMICS taxa. We also created a custom RefSeq-DB database comprised of 35 809 assemblies associated with the same 646 species (Table 1). Focusing only on nonensemble classifiers, Kraken2-HiTaxon-DB (0.683 MCC) performed the best among the classifiers (Fig. 5A). In addition, when compared to these subsets of individual classifiers, Kraken2-HiTaxon-DB predicted the most reads to at least one taxonomic rank (Table 4). Extending our analyses to ensemble algorithms, we found that further improvement in species classification could be derived from using Kraken2-HiTaxon-Align, the hierarchical ensemble of Kraken2 and BWA (0.737 MCC). Kraken2-HiTaxon-Align was also found to be the best performing classifier in terms of recall (0.741) and precision (0.998) (Supplementary Data S5). When compared to the baseline Kraken2-RefSeq-DB, the Kraken2-HiTaxon-Align ensemble resulted in a 22.72% improvement in species MCC. The second dataset, Mock Community 2, comprised 2 599 072 paired-end and single-end reads from a transcriptomic analysis of human bacterial pathogens (Avican *et al.* 2021). Classifiers were generated using 10 863 883 HiTaxon curated sequences from 9369 assemblies (1275 species) that pertain to 23 genera associated with the 28 species in Mock Community 2. Like previous, we also created a RefSeq-DB database composed of 100 681 RefSeq assemblies (389 602 189 coding sequences) associated with the same 1275 species. Again, Kraken2-HiTaxon-DB (0.569 MCC) performed the best among the individual classifiers in species prediction (Fig. 5B), while also predicting the most reads to at least one taxonomic rank. (Table 5). Similarly, the Kraken2-HiTaxon-Align ensemble provided the best performance amongst all classifiers (0.632 MCC), representing a 19.2% improvement in species classification relative to the baseline Kraken2-RefSeq-DB algorithm. From a recall and precision perspective (Supplementary Data S6), while Kraken2-HiTaxon-Align had the highest recall (0.621), its precision was 0.0036 lower then Kraken2-HiTaxon-DB. Interestingly, amongst all ensembles, Kraken2-HiTaxon-Align relied the least on its primary classifier, Kraken2, for species classification in both Mock Community 1 and 2. Instead, the majority of its predictions were derived from BWA and its genus-specific indices.

**Table 4.** Contribution of primary classifiers and specialized classifiers in ensembles for species classification of 4 556 549 Mock Community 1 reads.

Classifier^a^	No. of reads classified at species-level	% Contribution by primary classifier	% Contribution by specialized classifiers	No. of reads classified at any level
LCL-LCPN	3 489 839	NA	NA	4 545 554
Kaiju-HiTaxon-DB	2 140 521	NA	NA	3 752 240
Kaiju-RefSeq-DB	1 686 345	NA	NA	3 680 198
Kraken2-HiTaxon-DB	3 221 871	NA	NA	4 018 233
Kraken2-RefSeq-DB	2 714 535	NA	NA	4 005 581
Kraken2-HiTaxon-ML	3 867 946	9.64	90.36	4 018 233
Kaiju-HiTaxon-ML	3 466 138	7.54	92.46	3 752 240
Kraken2-HiTaxon-Align	3 951 427	0.04	99.96	4 018 233
Kraken2-Kaiju-Ens	3 272 554	98.45	1.55	4 024 934

a Kraken2-Kaiju-Ens does not have multiple specialized classifiers, and instead uses species predictions from Kaiju-HiTaxon-DB only when Kraken2-HiTaxon-DB does not have a species prediction (see Section 2 for more details). For clarity, we refer to Kraken2 as the primary classifier and Kaiju as the specialized classifier in this instance.

**Table 5.** Contribution of primary classifiers and specialized classifiers in ensembles for species classification of 2 599 072 Mock Community 2 reads.

Classifier^a^	No. of reads classified at species-level	% Contribution by primary classifier	% Contribution by specialized classifiers	No. of reads classified at any level
LCL-LCPN	1 453 937	NA	NA	2 599 042
Kaiju-HiTaxon-DB	760 393	NA	NA	1 750 060
Kaiju-RefSeq-DB	911 832	NA	NA	1 950 912
Kraken2-HiTaxon-DB	1 443 951	NA	NA	2 122 577
Kraken2-RefSeq-DB	1 308 778	NA	NA	2 110 559
Kraken2-HiTaxon-ML	1 922 173	6.38	93.62	2 122 577
Kaiju-HiTaxon-ML	1 498 826	4.25	95.75	1 950 912
Kraken2-HiTaxon-Align	1 993 052	0.12	99.88	2 122 577
Kraken2-Kaiju-Ens	1 497 433	96.42	3.58	2 125 372

a Kraken2-Kaiju-Ens does not have multiple specialized classifiers, and instead uses species predictions from Kaiju-HiTaxon-DB only when Kraken2-HiTaxon-DB does not have a species prediction (see Section 2 for more details). For clarity, we refer to Kraken2 as the primary classifier and Kaiju as the specialized classifier in this instance.

When evaluating the runtime of HiTaxon-based classifiers for both Mock Community 1 and 2 on the SciNet operated Niagara compute cluster (Table 6), we found that the two independent classifiers, Kraken2-HiTaxon-DB and Kaiju-HiTaxon-DB, were the two fastest approaches. Comparing hierarchical ensembles, we observed that using Kraken2-HiTaxon-Align substantially reduced the runtime when compared to either Kraken2-HiTaxon-ML or Kaiju-HiTaxon-ML. Runtimes for evaluating reads in Mock Community 1 and 2 were also measured on a 2.6 GHz 6-core Intel Core i7 MacBook Pro with 16 GB of RAM.

**Table 6.** Runtime of classifiers using HiTaxon curated sequences on Mock Community 1 and 2.

Classifier	Mock Community 1		Mock Community 2	
	SciNet runtime	MacBook runtime	SciNet runtime	MacBook runtime
LCL-LCPN	26 min and 44 s	4 h and 21 min	24 min and 9 s	3 h and 54 min
Kaiju-HiTaxon-DB	49 s	3 min and 9 s	31 s	3 min and 57 s
Kraken2-HiTaxon-DB	4 s	1 min and 6 s	6 s	2 min and 33 s
Kraken2-HiTaxon-ML	11 min and 59 s	3 h and 39 min	11 min and 47 s	3 h and 11 min
Kaiju-HiTaxon-ML	11 min and 42 s	3 h and 32 min	11 min and 8 s	3 h and 2 min
Kraken2-HiTaxon-Align	9 min and 24 s	14 min and 4 s	5 min and 34 s	18 min and 29 s
Kraken2-Kaiju-Ens	2 min and 43 s	6 min and 33 s	1 min and 34 s	8 min and 21 s

## 4 Discussion

Metagenomics and metatranscriptomics represent powerful technologies to functionally interrogate microbiomes (Chung *et al.* 2020). A key challenge is classifying sequenced reads to their taxa of origin. This is of particular concern for metatranscriptomics applications, typically composed of tens or even hundreds of millions of reads that are required to construct an accurate profile of gene expression for complex microbial communities. In the absence of paired metagenomics datasets, which can increase project costs, researchers are reliant on dedicated short read classifiers. Here, we present HiTaxon, a framework for generating hierarchical ensembles to improve species prediction. A core benefit of HiTaxon is the automated generation of nonredundant coding sequences for database construction. We found with few exceptions, that the use of these custom databases with fewer redundant sequences enhanced species-level predictions, functioning as a potential solution to concerns pertaining to the effects of large databases on read classification (Nasko *et al.* 2018, Smith *et al.* 2022). In addition, HiTaxon databases generated fewer false positives relative to databases composed of unprocessed RefSeq sequences. Thus, HiTaxon more effectively mitigates previously reported concerns regarding the susceptibility of reference-dependent classifiers with custom databases to generating false positives (Marcelino *et al.* 2020). Furthermore, with the current RefSeq database occupying over 1 TB of storage and requiring several days to build for reference-dependent tools (Wright *et al.* 2023), HiTaxon significantly reduces the computational overhead for taxonomic classification through reducing redundant data.

Given that taxonomic categorization involves organizing organisms into distinct hierarchical groups based on evolutionary relationships (Ereshefsky 1997), we investigated the use of hierarchical ML ensembling methods for species classification of short reads. We determined that our LCL-LCPN strategy provided improved species-level performance relative to other hierarchical approaches. Key to settling on the structure of our hierarchical ensemble was the finding that errors compounded from misassignments at higher taxonomic ranks outweighed the benefit of using hierarchy-informed predictions across all taxonomic ranks. Rather, limiting hierarchy-informed predictions to lower taxonomic ranks yielded superior performance. While Kraken2 continues to lead the field in taxon assignments, we found its performance could be enhanced through a hierarchical ensembling strategy where Kraken2’s genus-level predictions were supplemented with specialized classifiers operating at the species-level. Testing of Kraken2-HiTaxon hierarchical ensembles using simulated and experimental datasets, demonstrated the best species-level assignments in all instances. To the readers, we recommend using the Kraken2-HiTaxon-Align ensemble, as it had both the highest MCC and recall alongside a top two precision relative to other classifiers. It also required a lower runtime when compared to ensembles with ML classifiers.

Although HiTaxon offers considerable advantages, we do note several caveats. One important weakness is that the hierarchical ensembles are slower than the independent classifiers during evaluation. While one solution is to use the independent Kraken2-HiTaxon-DB classifier, future efforts should be made to optimize HiTaxon hierarchical algorithms to reduce the trade-off between maximizing species performance and runtime. Another key weakness, relative to the other classifiers with custom RefSeq-DB databases, is that the use of HiTaxon’s nonredundant sequences resulted in a marginal decrease in performance at higher taxonomic ranks. Future design improvements should therefore focus on HiTaxon’s data collection and processing pipeline, such that performance at higher ranks is improved whilst avoiding compromising the accuracy of predictions at the species-level. Further, since RefSeq is historically biased in taxa associated with medical, industrial or agricultural applications, alternative resources such as collections of MAGs hosted by, e.g. the MGnify resource (Richardson *et al.* 2022), offer potential for generating more appropriate environment-specific classifiers. In addition, whilst the development of HiTaxon was focused on the task of predicting the taxonomic origin of short reads, extending HiTaxon to function on longer reads would be useful as a recent simulation study highlighted that using longer reads from Nanopore sequencing improved classification (Govender and Eyre 2022). In a similar vein, for instances in which only short reads are available, developing a variant of HiTaxon which could be used on assembled contigs would be useful as these assemblies have been found to improve species identification (Tran and Phan 2020). With respect to the ML component of the mixed reference-dependent and ML hierarchical ensembles, additional exploration of hyperparameters could further enhance performance, as hyperparameters derived for the human conjunctival microbiome simulation were used throughout this study. Beyond hyperparameters, alternative ML algorithms should continue to be explored. For example, with demonstrated benefits in other domains within biology, fine-tuning transformers pre-trained on sequence data for species classification represents an alternative avenue worth further exploration (Ji *et al.* 2021). Similarly, for hierarchical ensembles composed entirely of reference-dependent classifiers, the integration of alternative tools would be interesting to explore.

## Supplementary Material

vbae016_Supplementary_Data

## Data Availability

The software is freely available under the GNU public license V3 and can be accessed at: https://github.com/ParkinsonLab/HiTaxon. In addition, test files used to compare classifiers in the study can be found at: https://doi.org/10.5281/zenodo.8335901.
